# Year-Book of the Scientific and Learned Societies of Great Britain and Ireland

**Published:** 1892-12

**Authors:** 


*Year-Book of the Scientific and Learned Societies of Great Britain and Ireland.* London: C. GRIFFIN & CO. 1892.

This is the ninth annual issue of a useful chronicle of the work done by various societies, compiled from data published by the Societies themselves.

We fail to see on what principle our own Bristol Medico-Chirurgical Society should be mentioned only on the last page, in the smallest of type, and under the heading "Other Medical Societies."

